# 107 Meta-Analysis of Clinical and Preclinical Microbiome Data from Studies of Burn Injury

**DOI:** 10.1093/jbcr/irad045.080

**Published:** 2023-05-15

**Authors:** Timothy Horseman, Andrew Frank, Jeffrey Shupp, David Burmeister

**Affiliations:** Uniformed Services University of the Health Sciences, Bethesda, Maryland; Uniformed Services University of the Health Sciences, Bethesda, Maryland; Firefighters' Burn and Surgical Research Laboratory, MedStar Health Research Institute, Georgetown University School of Medicine, Washington, DC; Uniformed Services University of the Health Sciences, Bethesda, Maryland; Uniformed Services University of the Health Sciences, Bethesda, Maryland; Uniformed Services University of the Health Sciences, Bethesda, Maryland; Firefighters' Burn and Surgical Research Laboratory, MedStar Health Research Institute, Georgetown University School of Medicine, Washington, DC; Uniformed Services University of the Health Sciences, Bethesda, Maryland

## Abstract

**Introduction:**

Following burn injury, the ecology of the host microbiota is disrupted to varying degrees and may leave the patient susceptible to opportunistic pathogens or disrupt the endogenous flora needed to potentiate recovery and homeostasis. Studies examining the microbiome post-burn injury usually have a limited sample size which, coupled with experimental and analysis variation, impacts overall interpretation of data.

**Methods:**

We performed a meta-analysis of publicly available sequencing data from burn patients and burn animals to determine if there were consistent alterations in the microbiome across various anatomical sites and species. A MEDLINE search was used to identify applicable publications. Corresponding authors of papers without available data were contacted, and data were used if a response occurred. Ten human and animal 16S rRNA sequencing studies spanning respiratory, urinary, cutaneous, and gastrointestinal microbiomes were included. Raw sequencing data were systematically analyzed with taxonomic classification and α and β diversity metrics generated.

**Results:**

Alpha diversity was consistently lower post-burn, except for human skin (Figure 1) due to perianal skin sampling of burn patients resulting in higher species richness than controls. Weighted UniFrac distance analysis showed that rodent specimens clustered less closely to humans than pig samples (*P* < 0.0001) for both rectal and skin samples. Host species (R2 = 0.21, *P* = 0.001), and institute (R2 = 0.35, *P* = 0.001) had a significant impact on the β diversity. In rectal samples, bacterial composition in pig and human burn samples included Bacteroidetes, Firmicutes, and Proteobacteria, while rodent samples were dominated by over 90% Firmicutes. Burns induced an increase of Proteobacteria and a decrease in Bacteroidetes and Firmicutes in pig rectal samples. Proteobacteria and Firmicutes increased on burned skin in each host species. Longitudinal studies revealed a decrease in α diversity upon burn injury with β diversity and taxa shifts.

**Conclusions:**

Overall, our results suggest that host species and the sequencing site strongly influence microbiome structure. Burn-induced alterations in microbiome diversity and taxa exist across hosts, with certain metrics more influenced by host.

**Applicability of Research to Practice:**

Coordinated, multi-center studies, both clinical and pre-clinical, are needed to truly realize the diagnostic and therapeutic potential of the microbiome for improving outcomes post-burn.

